# Can the use of a next generation partograph based on WHO’s latest intrapartum care recommendations improve neonatal outcomes? (PICRINO) Study protocol for a stepped-wedge cluster randomized trial

**DOI:** 10.1371/journal.pone.0316336

**Published:** 2025-03-04

**Authors:** Anna Ramö Isgren, Radha Korsoski, Thomas Abrahamsson, Sophia Brismar Wendel, Karin Källén, Louise Lundborg, Katarina Remaeus, Kristin Thomas, Anna-Karin Wikström, Ylva Carlsson, Marie Blomberg

**Affiliations:** 1 Department of Obstetrics and Gynecology, Linköping University, Linköping, Sweden; 2 Department of Biomedical and Clinical Sciences, Linköping University, Linköping, Sweden; 3 Region Västra Götaland, Department of Obstetrics and Gynecology, Sahlgrenska University Hospital, Gothenburg, Sweden; 4 Department of Obstetrics and Gynecology, Institute of Clinical Sciences, Sahlgrenska Academy, University of Gothenburg, Gothenburg, Sweden; 5 Crown Princess Victoria Children’s Hospital, Linköping, Sweden; 6 Department of Obstetrics and Gynecology, Danderyd Hospital, Stockholm, Sweden; 7 Department of Clinical Sciences, Karolinska Institutet, Danderyd Hospital, Stockholm, Sweden; 8 Center for Reproductive Epidemiology, Tornblad Institute, Lund University, Lund, Sweden; 9 Clinical Epidemiology Division, Department of Medicine, Solna, Karolinska Institutet, Stockholm, Sweden; 10 Department of Health, Medicine and Caring, Medical Faculty, Linköping University, Linköping, Sweden; 11 Department of Obstetrics and Gynecology, Uppsala University Hospital, Uppsala, Sweden; 12 Department of Women’s and Children’s Health, Uppsala University, Uppsala, Sweden; 13 Centre of Perinatal Medicine and Health, Institute of Clinical Sciences, Sahlgrenska Academy, University of Gothenburg, Gothenburg, Sweden; PLOS: Public Library of Science, UNITED KINGDOM OF GREAT BRITAIN AND NORTHERN IRELAND

## Abstract

**Background:**

Monitoring labor and childbirth, early recognition, and treatment of complications are critical for preventing adverse birth outcomes. However excessive use of interventions during labor has not been demonstrated to enhance subsequent birth outcomes and may, in fact, pose a risk of harm. The World Health Organization has recently synthesized research from the last decade concerning labor progress and patient-centered care into a new recommendation: the Labour Care Guide (LCG). No trial has, however, compared the LCG with standard care regarding adverse neonatal outcomes or the degree of safety associated with implementing this recommendation within a high-resource setting, and its potential to enhance birth outcomes remains undetermined.

**Aim and hypothesis:**

This trial aims to evaluate the impacts of using two different guidelines for monitoring labor with respect to neonatal and maternal outcomes, the LCG and the currently used standard care guideline. The hypothesis is that use of the LCG will reduce the number of adverse neonatal outcomes and decrease the rate of intrapartum cesarean sections, as compared with standard care.

**Materials and methods:**

A national, multicenter, stepped-wedge cluster randomized controlled trial that includes 24 Swedish maternity wards randomized to six clusters. The trial is planned to run during a 22 month period in 2023–2025 and the intervention LCG will be implemented in the six clusters, with three months intervals. The recruited wards will together have approximatively 100,000 births during the study period. Outcome data will be extracted from the Swedish national pregnancy, neonatal, and patient registers. Two safety analyses will be performed at one-third and two-thirds of the way through the trial.

**Discussion:**

The LCG offers a promising new approach, but its effectiveness and safety in high resource settings remain unexplored and must be studied further before LCG can be fully implemented in settings with similar health care.

**Trial registration:**

The trial has been registered at www.clinicaltrials.gov: NCT05560802.

## Introduction

Worldwide, there are ongoing discussions regarding how surveillance and management of labor is best performed to optimize outcome for both the mother and the neonate. Current guidelines regarding labor progress are based on research conducted by Friedman in the 1950s, with minor adjustments made in the 1990s [1]. Since then, the current definition of normal labor progress and guidelines for labor progress have been challenged [2–4]. In 2018, the World Health Organization (WHO) published updated recommendations on intrapartum care for a positive childbirth experience. The recommendations included new definitions of onset and progress of labor and high-lighted the importance of woman-centered care [5]. In 2020 a new tool for structured documentation and implementation of the recommendations, the Labour Care Guide (LCG) was published based on the new recommendations [5–7]. The LCG was designed to ensure evidence-based care reflecting new evidence, both regarding labor progress but also supportive care and shared decision making. The LCG entails increased possibility of individualized care in active labor with focus on ensuring safety, avoiding unnecessary interventions and providing supportive care [8].

The LCG was developed in low- and middle-resource settings, mainly for women with low-risk pregnancies, but WHO recommends use of the LCG for all pregnancies and in all formal healthcare settings. Differences between the LCG and standard care guidelines in Sweden are shown (Table 1). The usability and feasibility of the LCG have been tested in health facilities in low- and middle-resource settings in South America, Asia, and Africa, but no study has compared the LCG with standard care regarding adverse neonatal outcomes, neither in these settings nor in high-resource settings [9]. Introducing the LCG in Sweden as a randomized controlled trial, *Can the use of a next generation Partograph based on WHO’s latest Intrapartum Care Recommendations Improve Neonatal Outcomes?* the PICRINO trial will contribute with fundamental knowledge of how the LCG will affect different key outcomes of labor in a high-resource setting.

**Table 1:** Differences between the labour care guide (LCG) and standard care in Sweden.

LCG	Standard care
Active phase of labor: 5 to 10 cm of cervical dilatation	Active phase of labor: 3 to 10 cm of cervical dilatation
Evidence-based time limits, at each centimeter of cervical dilatation	Fixed 1 cm cervical dilation per hour.
Meticulous monitoring of the second stage of labor	Local initiatives but no uniform recommendations
Explicit recording of supportive care on a continuous basis	Local initiatives but no uniform recommendations
Shared decision-making is highlighted, and has its own section for documentation	Not highlighted and no specific section for documentation
All deviations are highlighted, and a corresponding plan is required from the provider	No system available to highlight deviations

### The LCG

The WHO recommendations on intrapartum care for a positive childbirth experience are based on evidence from systematic reviews. The WHO concluded that older partographs, especially those featuring ‘alert’ and ‘action’ lines, which are currently utilized in many high-resource countries, are no longer scientifically valid [10,11]. Furthermore, many partographs in current use do not monitor supportive care interventions such as companionship, the woman’s mobility, birth position, intake of fluid, or use of pain relief.

To support the implementation of these recommendations, the WHO developed a “next generation” partograph called the LCG, along with a comprehensive LCG user manual. The LCG is a labor tool consisting of seven sections which supports providers to effectively monitor and document supportive care, maternal and fetal status, labor progress, shared decision-making, and it offers timely reminders on appropriate clinical and supportive care. The LCG aims to promote patient-centered care, stimulate providers to think critically around labor decision-making, and individualize labor monitoring [6].

### The Swedish labour care guide (LCG-SE)

The Swedish version of LCG, called LCG-SE, has been systematically developed by health care professionals in the delivery ward of Linköping University Hospital, Sweden. The aim was to systematically modify and adapt a version of the WHO’s LCG to a Swedish setting, to be used in the PICRINO trial. A PICRINO Group, including obstetricians, midwives, and assistant nurses, was established to develop the Swedish version of the LCG and in the next step educate delivery wards included in the PICRINO trial in the LCG. The LCG manual was translated into Swedish and structured workshops with the healthcare professionals working in the delivery ward in Linköping were conducted. Revision of the LCG tool, based on input from the workshops, was made aiming to identify elements to adapt. The blueprint of a modified protocol was tested by case discussions by both local and national clinicians, resulting in some minor further adjustments. These adaptations included adding cardiotocography to assess fetal status, ensuring continuous staff attendance, and eliminating the surveillance of molding and proteinuria. Furthermore, the Swedish version was developed to be used as a digital tool instead of a paper-based tool. In total, the final version of the LCG-SE is a moderately modified version of the original LCG. No changes have been made in the clinical recommendations embedded within the LCG. Hereafter, in this manuscript, we will use the abbreviation LCG instead of LCG-SE for simplicity and readability.

### Aim

The aim of this trial is to evaluate the impacts of using two different guidelines (the LCG and the currently used standard care guideline) for monitoring labor with respect to neonatal and maternal outcomes. The hypothesis is that the use of the LCG will reduce adverse neonatal outcomes and decrease the rate of intrapartum cesarean sections (CSs) compared with the use of standard care. Additionally, childbirth experience, health economy and perinatal interventions and complications, will be compared between the LCG and standard care.

## Materials and methods

### Study design

**Fig 1:**
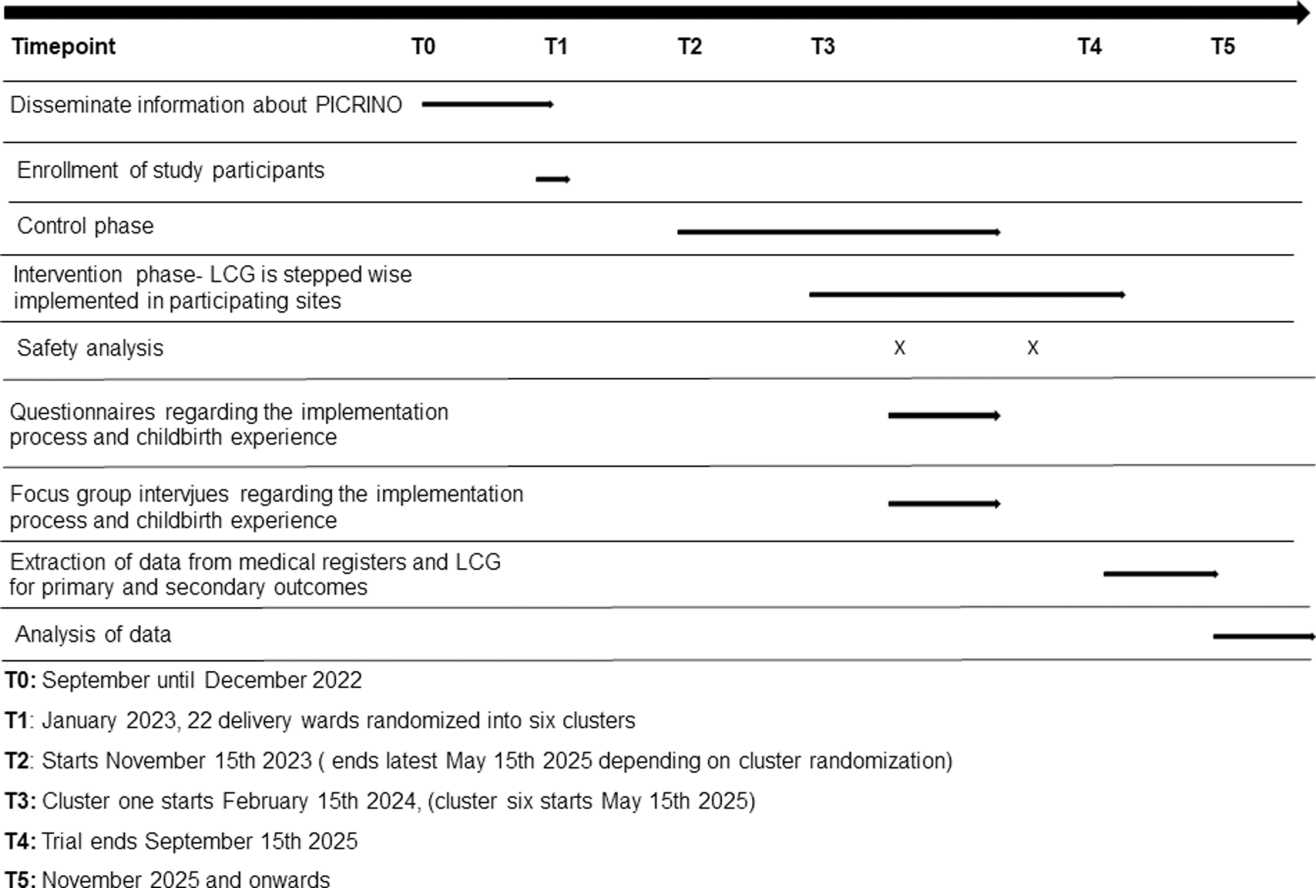
Schedule for enrollment, intervention and assessments.

This trial is a national, multicenter, stepped-wedge cluster randomized trial including 24 Swedish delivery wards randomized to six clusters [12]. The trial is planned to run during a 22-month period in 2023-2025. The intervention LCG will be implemented in the six clusters, with three months intervals, over a 19-month period (February 15th 2024 until September 15th 2025). The population, intervention, comparison, and outcome (PICO) are presented (Table 2) and the schedule for enrollment, intervention and assessments is presented (Fig 1).

**Table 2:** The population, intervention, comparison, and outcome (PICO).

**Population**	All women in active labor at 24 participating delivery wards in Sweden in the period of 15th November 2023 through 15th September 2025
**Intervention**	Use of the LCG, implemented at the participating delivery wards using a stepped-wedge cluster randomized design.
**Comparison**	Use of standard care (partograph and labor guidelines).
**Outcome**	*Primary*: 1) a composite neonatal outcome set; and 2) the rate of intrapartum cesarean section. *Secondary/exploratory*: neonatal and maternal outcomes, women’s and partners’ childbirth experience, obstetric staffs’ experience of the LCG, health economic evaluation.

### Recruitment and randomization

**Fig 2:**
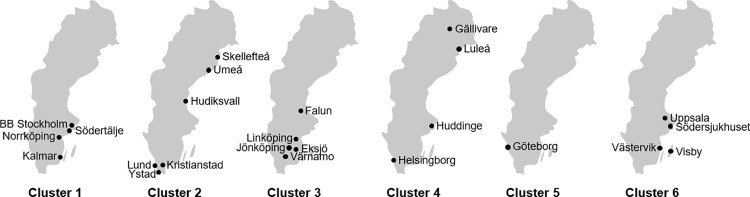
Participating delivery wards randomized into six clusters.

All 44 delivery wards in Sweden received a first invitation to participate in the PICRINO trial in 2022. The recruitment process was stopped in December 2022 when 24 delivery wards had been contracted for participation in the full trial. Five additional wards are currently on a waiting list, to provide cover for potential dropouts. Participating delivery wards exhibit a range of sizes and encompass university hospitals, county hospitals, and district hospitals. The delivery wards were randomized into six clusters stratified by unit size. The geographic distribution of participating delivery wards in each cluster is shown (Fig 2).

First, each cluster will start in the control period. After the introduction of the LCG intervention data will be compared with data acquired during the ‘control phase’, i.e., the period during which standard care is followed, from the trial start to the time of introduction of the intervention. A transition period of two months between the introduction of the intervention and data outcome collection will be used. Outcome data during the transition time will be handled separately.

### Intervention

**Fig 3:**
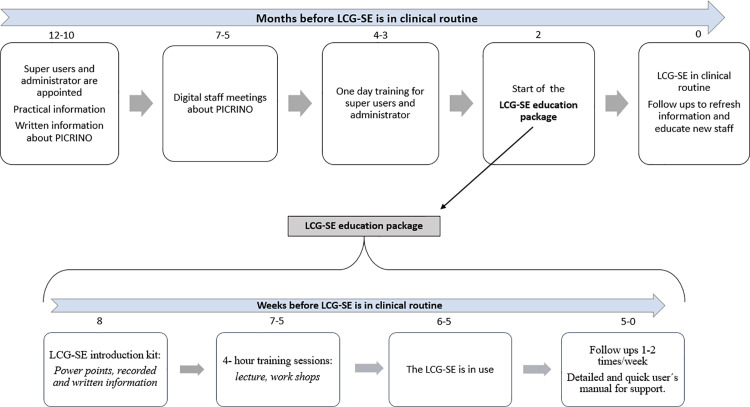
The implementation process for the LCG.

All included sites will undergo a structured implementation process with the LCG provided by the PICRINO Group, including on-site education of all staff (Fig 3). Each site will have 2-6 selected local super-users (midwives and/or obstetricians) for local support at their site and they will have regular contact (phone calls, e-mails, online meetings) with the PICRINO Group during the implementation process. Information about the trial and the education package (power points, recorded and written information) will be provided at a password protected page at the website PICRINO.se. Each site will also have local appointed administrative staff to facilitate the administrative work that the implementation entails. Due to the nature of the intervention it is not possible to blind the participants.

### Variables and measures

The trial has two primary outcomes: a composite neonatal outcome set and the rate of intrapartum CS. The primary outcomes and the secondary outcomes are further described (Table 3).

**Table 3:** Primary and secondary/exploratory outcomes.

Primary outcomes
1. A composite neonatal outcome set comprising perinatal and neonatal mortality, 5-minute Apgar score <7, occurrence of hypoxic ischemic encephalopathy II-III, and admission to a neonatal unit 2. The rate of intrapartum cesarean sections
**Secondary and exploratory outcomes**
**Secondary**
women’s and partners’ childbirth experience, obstetric staffs’ experience of the LCG, costs and resource requirements (diagnosis and interventions, cost per patient, occupancy rate, staff density)
**Exploratory**
***Neonatal*:** Variables involve the components of the primary composite neonatal outcome set will be studied individually (neonatal mortality, 5-minute Apgar score <7, occurrence of hypoxic ischemic encephalopathy II-III, admission to a neonatal unit), intracranial hemorrhage, seizure, meconium aspiration syndrome, 5-minute Apgar score <4, respiratory disorder, infection, hypoglycemia, treated jaundice, shoulder dystocia, obstetric brachial plexus injury
***Maternal*:** spontaneous vaginal delivery, instrumental delivery, artificial rupture of membrane, oxytocin use for augmentation, epidural use, postpartum hemorrhage >1000 ml, perineal laceration (grade II-IV), indications for intrapartum cesarean section, cervical dilation at onset of augmentation and time estimates of the active phase of labor and the 2nd stage of labor

The rationale for having two primary outcomes is that if an intervention (the LCG) improves one outcome (neonatal outcome) at the expense of an increased frequency of interventions (CS), one should critically appraise whether the LCG should be introduced.

All primary outcome variables in the present trial are consistent with the proposed Swedish Perinatal Core Outcome Set, except the Apgar score, for the management of labor at or near term (SPeCOS) [13].

### Data collection

Outcome data will be extracted from the Swedish Pregnancy Register (SPR), the Swedish Neonatal Quality Register (SNQ), and the Swedish National Patient Register. The registries are described in detail (Table 4). A PICRINO database, which contains the trial specific electronic case report form, has been established via the company OMDA. OMDA also runs the digital platform for the SPR and SNQ.

**Table 4:** Swedish quality registries used for the PICRINO trial.

**The Swedish pregnancy register**
A certified national quality register, initiated by the Swedish healthcare regions, combines prospectively collected data from early pregnancy to two months after birth. This includes data from the Swedish Maternal Health Care Register, the Swedish National Quality Register for Prenatal Diagnosis, and electronic, standardized prenatal, delivery, and neonatal records. The register includes more than 95% of all deliveries in Sweden and covers pregnancies from the first antenatal care visit until the follow-up visit at 8–12 weeks postpartum. It contains information on maternal characteristics, medical and reproductive history, pregnancy examinations, delivery outcomes and follow-up [14].
**The Swedish neonatal quality register**
A national quality register for neonatal care. All neonatal departments in Sweden report prospectively standardized data on admitted infants including basic information about pregnancy and childbirth, as well as the condition, treatment and diagnoses of the infant during the neonatal period according to the Swedish version of International Classification of Diseases 10th revision (ICD-10), as well as information from follow-up visits at 2 and 5.5 years of age [15].
**The national patient register**
A mandatory health register including diagnoses on hospital admissions and outpatient visits in specialist care. Information will be retrieved on ICD-10 diagnoses and interventions for women during pregnancy and the postpartum period, chronic or previous disease in the mother as well long-term follow-up of their children [16].

Data from the different registries, the electronic case report form, and the questionnaires will be linked through the personal identification number and subsequently replaced by a study ID.

### Data collection of secondary outcomes (not available in the described registers)

#### Childbirth experience (substudy Ia).

Four weeks postpartum questions about birth experience will be randomly distributed to women at three to four participating sites with different levels of care. Women will be informed and invited to participate during follow-up visits at the maternity wards. After electronic (using the Swedish Bank-ID) informed consent is obtained women will be linked to the validated Childbirth Experience Questionnaire version 2 (CEQ2) [17]. The CEQ2 comprises 22 items aggregated into 4 domains, i.e., Own capacity, Professional support, Perceived safety, and Participation. The total CEQ scores, as well as scores for all four domains will be compared between women giving birth during standard care versus LCG.

#### Childbirth experience (substudy Ib).

For the individual interviews, a purposive sampling strategy will be used to recruit a broad range of women based on age, parity, number of previous births, geographic location, and socioeconomic status. Women will be informed and invited to participate during follow-up visits at the maternity wards. Informed consent will be obtained verbally prior to each interview.

All interviews will take place 2–3 months postpartum. An interview guide including open-ended questions to explore perceptions and experiences of childbirth will be used. Follow-up questions and probes will also be used to clarify and deepen the understanding of the participants’ responses. We estimate that approximately 20–25 interviews will be adequate to capture the study aim based on the research question and analysis method.

To further explore childbirth experiences interviews with partners will be carried out. For the partners, the recruitment strategy, eligible criteria, data collection and analysis methods will follow the same procedures as the interviews with the women.

#### Provider experience (substudy IIa).

Provider perception of organizational readiness for implementing LCG will be measured during the transition period and further adherence to the tool will be evaluated. After the introduction, where all staff have received training about LCG, the validated tool E-Ready, will be e-mailed by a web-link to nurse assistants, midwives and physicians at selected study sites [18]. E-Ready consists of 6 sections with sub items investigating perceived conditions for change at workplace, perceived individual conditions for change, perceived support and engagement among management, perceived readiness among colleagues, perceived consequences on status quo and perceived workplace attitudes. Informed consent to distribute questionnaires will be obtained from each hospital administrator and written participant information will be sent along with the questionnaire to the providers.

#### Provider experience of the LCG (substudy IIb).

Providers will be invited to take part in focus group interviews via e-mail by a web-link which also include information about the study. Focus group interviews are deemed appropriate since the implementation of LCG is team based and this method would enable inter-professional interaction and discussion about implementation experiences. We estimate that 4-6 focus groups with about six informants per group will be sufficient to capture rich data on implementation. Written informed consent will be made at the focus group, prior discussion and recording starts. A semi-structured interview guide will be employed and aim to capture experiences, perceptions and expectations of LCG as well as hinders and facilitators for implementation.

#### Provider experience of the LCG (substudy IIc).

A questionnaire evaluating usability and satisfaction among health care providers will be used. The questionnaire will be sent to enrolled clinics and their health care personnel working at the labour ward about four months after the clinic has started using the LCG-SE. Midwifes, obstetricians and auxiliary nurses) will be invited to answer the questionnaire. The questionnaire used is based on two questionnaires developed by Vogel et al [9].

#### Economic considerations (substudy III).

Data on health care utilization for both women and children, during labor but also during the following year will be collected. Data on healthcare utilization, DRG and cost per patient are obtained from each region’s patient register and will be interlinked with data from the SPR and SNQ. The introduction of LCG may affect the time in labor and thus the logistics of how providers and rooms need to be scheduled. In order to analyze the effects from a logistic perspective, the occupancy rate and provider structure at each unit will be collected.

### Ethics

The PICRINO trial was approved by the Swedish Ethical Review Authority on November 9, 2022, (Dnr. 2022-04868-01) and five amendments were approved on July 19, 2023 (Dnr. 2023-04264-02), on October 4, 2023 (Dnr 2023-05656-02), on April 23, 2024 (Dnr 2024-02175-02), on May 17, 2024 (Dnr. 2024-02147-02), and on June 12, 2024 (Dnr 2024-03265-02). Since the study aims to examine the effect of implementing a new guideline, the ethics committee determined that no information to or informed consent from participants was required for the main study. For the sub studies including women’s and partners’ childbirth experience and obstetric staffs’ experience information is given and informed consent is obtained. Any changes to the protocol which may affect the conduct of the study, decrease benefits or increase risk of harm to the participants will be communicated to the ethics committee for review and approval.

### Statistical analyses

#### Estimated sample size and power.

Power analyses have been performed considering the number of time periods, number of clusters, the number of observations per time-period, the before-trial incidence of outcome, the between-units variation coefficient, and the anticipated risk reduction. The incidence of any adverse neonatal outcome (defined as perinatal or neonatal death, Apgar score < 7 at 5 minutes, occurrence of hypoxic ischemic encephalopathy grade II-III, or admission to neonatal unit) was estimated using data from the SPR and the SNQ 2017-2021.

Number of time periods: All births during a period of two years divided into seven time periods. Number of clusters: Half of the 44 Swedish delivery wards will participate in the trial (thus, 22 units). Number of observations per time period: The total number of births that met the inclusion criteria during the five observed years was 466 615, giving a yearly estimate of 93 300 births that meet the inclusion criteria. Based on the number of deliveries during 2021, the 24 recruited units will together have approximatively 100 000 births during a two-year period, which corresponds to approximately 14 000 births per time unit. Pre-trial mean incidence of composite outcome: 6.73%. Between-unit coefficient of variation: 0.46 (variance between units/ overall mean). Anticipated risk reduction: 20% risk reduction (corresponding to a risk ratio of 0.8). With a significance level of 0.05, the power to detect the anticipated risk reduction would be greater than 0.999 given the settings listed above.

The mean rate of intrapartum CS for term pregnancies in Sweden between 2017 and 2021 was 9.1% (range between units, 5.6%-11.8%). No power analysis was performed explicitly for intrapartum CS (due to the higher occurrence of intrapartum CS compared to the incidence of the composite neonatal outcome). If adjusting for multiple comparisons, considering that the setting has two primary outcomes (thus instead of using alpha = 0.05, using alpha = 0.05/2 with corresponding Za = 2.24), the power to detect the anticipated risk reduction would still be satisfactory (0.993).

#### Safety analysis.

Two safety analyses will be performed at one-third and two-thirds of the way through the trial. The safety analyses will be performed by comparing the composite neonatal outcome set in the control group (standard care) and the intervention group (the LCG). Neonatal outcomes for the safety analyses will be extracted from the SPR and SNQ. A Data and Safety Monitoring Board (DSMB) will be established that includes a statistician, an experienced obstetrician, as well as a neonatologist. These individuals will all be independent of the PICRINO steering committee. The DSMB will have access to analysis/results and interpret the data. Stopping criteria is a statistically significant clinically worse outcome (p < 0.05) in the intervention group. The trial will not be interrupted due to a significantly better clinical outcome in the intervention group compared with the control group since the new guidelines could not be implemented faster than via the PICRINO trial protocol.

#### Statistical methods.

Generalized estimating equations (GEE) will be used to analyze the effect of the intervention, considering the individual-level binary outcomes. An exchangeable correlation structure will be assumed. The intervention effect will be expressed as relative risk, the after versus before intervention (=control) period. GEE analyses have been selected for this trial because they are more robust to variance mis-specification compared to Linear Mixed Models or General Linear Mixed Models. Although GEE can inflate type 1 errors when the number of clusters or time periods is low, this will not be a concern in the current trial setting [19,20]. The high number of clusters and time intervals will suit the asymptotic-based GEE. All analyses will be 2-sided, using a 5% significance level. A strict intention-to-treat policy will be applied, and all results will be reported according to the randomization scheme. Analyses will be performed using the SPSS (version 29), and R (version 4.2.2) software packages.

All interviews (individual and focus groups) will be audio recorded and transcribed verbatim. An explorative approach will be used employing inductive content analysis according to Elo and Kyngäs [21].

#### Quality assurance.

The trial sponsor is Marie Blomberg (MB). MB is responsible for the initiation, management and arranging the financing of the trial and carries the medico-legal responsibility associated with its conduct. All of Sweden’s county councils and regions are insured under Löf, the Swedish mandatory patient insurance.

The trial was registered at www.clinicaltrials.gov (NCT05560802) on September 29, 2022. Furthermore, the PICRINO trial is supported by the Swedish Network for National Clinical Studies in Obstetrics and Gynecology [22]. The final trial results will be published in peer-reviewed journals to be spread to a wider scientific audience and other relevant groups. The detailed study protocol is available at the website PICRINO.se.

#### Data management.

Confidentiality aspects such as data encryption and storage comply with the general data protection regulation. All data is stored in a secure online database provided by OMDA (www.omda.com), an international company specializing in web applications in the field of academic medicine.

#### Access to data.

Only those investigators who perform clinical duties at the involved hospital will have access to the original data via the participants’ electronic medical record. Once the data has been de-identified, all the investigators will have access to the data supplied by each participant in the electronic LCG tool.

## Discussion

The PICRINO trial is a national stepped wedge randomized cluster trial assessing the effect of the LCG on labor care compared with standard care. The expected major findings of this trial are a reduction of adverse neonatal outcomes and a reduced rate of intrapartum CSs. This could be attributable to the broader limits of normal labor progression, improved continuous support during labor, higher degree of individualized care, and high-lighted shared decision-making. Furthermore, we expect a higher degree of birth satisfaction among women and partners.

The stepped-wedge cluster, randomized design reduces the risk of selection bias (such as the exclusion of immigrants due to language barrier), while increasing the inclusion rate, feasibility, and generalizability of the results. Further each center contributes with both cases and controls, which can bridge the differences in clinical routines, as well as the differences in the proportions of high-risk pregnancies between participating sites. As the PICRINO trial includes more than half of eligible women in active labor in Sweden, the results could be generalized to women giving birth in similar settings, both nationally and internationally.

Limitations of the stepped wedge design include potential carryover effects from the exposure to the intervention, difficulty in blinding because both patients and assessors are aware of the step switch, logistical challenges in implementing the intervention sequentially, and the need for a longer study duration due to the staggered rollout of the intervention across clusters. There may also be unequal exposure to the intervention between the delivery wards and this challenge will be addressed through an extensive training program, which commenced during the implementation phase at Linköping University Hospital, where the LCG has already been piloted.

In conclusion, the LCG offers a promising new approach, but its effectiveness and safety in high-resource settings, including all birthing women, remains unexplored. It is of utmost importance to evaluate the implementation of new recommendations in terms of improved outcomes, costs, and women’s childbirth experience before implementation in full scale.

## Supporting information

S1 File SPIRIT checklist. (PDF)

S2 File Copy of the study protocol approved by the ethics committee. (PDF)

## References

[1] Friedman E. The graphic analysis of labor. Am J Obstet Gynecol. 1954;68(6):1568–75. doi: 10.1016/0002-9378(54)90311-7 13207246

[2] Oladapo OT, Souza JP, Fawole B, Mugerwa K, Perdoná G, Alves D, et al. Progression of the first stage of spontaneous labour: A prospective cohort study in two sub-Saharan African countries. PLoS Med. 2018;15(1):e1002492. doi: 10.1371/journal.pmed.1002492 29338000 PMC5770022

[3] Zhang J, Landy HJ, Ware Branch D, Burkman R, Haberman S, Gregory KD, et al. Contemporary patterns of spontaneous labor with normal neonatal outcomes. Obstet Gynecol. 2010;116(6):1281–7. doi: 10.1097/AOG.0b013e3181fdef6e 21099592 PMC3660040

[4] Lundborg L, Åberg K, Sandström A, Discacciati A, Tilden EL, Stephansson O, et al. First stage progression in women with spontaneous onset of labor: a large population-based cohort study. PLoS One. 2020;15(9):e0239724. doi: 10.1371/journal.pone.0239724 32976520 PMC7518577

[5] WHO. WHO recommendations. Intrapartum care for a positive childbirth experience. WHO guidelines approved by the guidelines review committee. Available from: https://www.who.int/reproductivehealth/publications/intrapartum-care-guidelines/en/2018 30070803

[6] WHO. WHO labour care guide user’s manual. Report No.: Licence: CC BY-NC-SA 3.0 IGO. Geneva: World Health Organization; 2020.

[7] Hofmeyr GJ, Bernitz S, Bonet M, Bucagu M, Dao B, Downe S, et al. WHO next-generation partograph: revolutionary steps towards individualised labour care. BJOG. 2021;128(10):1658–62. doi: 10.1111/1471-0528.16694 33686760 PMC9291293

[8] Vogel JP, Pujar Y, Vernekar SS, Armari E, Pingray V, Althabe F, et al. Effects of the WHO Labour Care Guide on cesarean section in India: a pragmatic, stepped-wedge, cluster-randomized pilot trial. Nat Med. 2024;30(2):463–9. doi: 10.1038/s41591-023-02751-4 38291297 PMC10878967

[9] Vogel JP, Comrie-Thomson L, Pingray V, Gadama L, Galadanci H, Goudar S, et al. Usability, acceptability, and feasibility of the World Health Organization Labour Care Guide: a mixed-methods, multicountry evaluation. Birth. 2021;48(1):66–75. doi: 10.1111/birt.12511 33225484 PMC8246537

[10] Abalos E, Oladapo OT, Chamillard M, Díaz V, Pasquale J, Bonet M, et al. Duration of spontaneous labour in “low-risk” women with “normal” perinatal outcomes: A systematic review. Eur J Obstet Gynecol Reprod Biol. 2018;223 123–32. doi: 10.1016/j.ejogrb.2018.02.026 29518643 PMC5884320

[11] Oladapo OT, Diaz V, Bonet M, Abalos E, Thwin SS, Souza H, et al. Cervical dilatation patterns of “low-risk” women with spontaneous labour and normal perinatal outcomes: a systematic review. BJOG. 2018;125(8):944–54. doi: 10.1111/1471-0528.14930 28892266 PMC6033146

[12] Hemming K, Haines TP, Chilton PJ, Girling AJ, Lilford RJ. The stepped wedge cluster randomised trial: rationale, design, analysis, and reporting. BMJ. 2015;350 h391. doi: 10.1136/bmj.h391 25662947

[13] Savchenko J, Asp M, Blomberg M, Elvander C, Hagman A, Pegelow Halvorsen C, et al. Key outcomes in childbirth: development of a perinatal core outcome set for management of labor and delivery at or near term. Acta Obstet Gynecol Scand. 2023;102(6):728–34. doi: 10.1111/aogs.14560 36965044 PMC10201975

[14] Stephansson O, Petersson K, Björk C, Conner P, Wikström A-K. The Swedish Pregnancy Register - for quality of care improvement and research. Acta Obstet Gynecol Scand. 2018;97(4):466–76. doi: 10.1111/aogs.13266 29172245 PMC5873375

[15] Norman M, Källén K, Wahlström E, Håkansson S, SNQ Collaboration. The Swedish Neonatal Quality Register - contents, completeness and validity. Acta Paediatr. 2019;108(8):1411–8. doi: 10.1111/apa.14823 31006126

[16] Ludvigsson JF, Andersson E, Ekbom A, Feychting M, Kim J-L, Reuterwall C, et al. External review and validation of the Swedish national inpatient register. BMC Public Health. 2011;11:450. doi: 10.1186/1471-2458-11-450 21658213 PMC3142234

[17] Dencker A, Taft C, Bergqvist L, Lilja H, Berg M. Childbirth experience questionnaire (CEQ): development and evaluation of a multidimensional instrument. BMC Pregnancy Childbirth. 2010;10:81. doi: 10.1186/1471-2393-10-81 21143961 PMC3008689

[18] Dannapfel P, Thomas K, Chakhunashvili A, Melin J, Trolle Lagerros Y. A self-help tool to facilitate implementation of eHealth initiatives in health care (E-ready): formative evaluation. JMIR Form Res. 2022;6(1):e17568. doi: 10.2196/17568 35037884 PMC8804954

[19] Hemming K, Taljaard M, McKenzie JE, Hooper R, Copas A, Thompson JA, et al. Reporting of stepped wedge cluster randomised trials: extension of the CONSORT 2010 statement with explanation and elaboration. BMJ. 2018;363:k1614. doi: 10.1136/bmj.k1614 30413417 PMC6225589

[20] Ford W, Westgate P. Maintaining the validity of inference in small-sample stepped wedge cluster randomized trials with binary outcomes when using generalized estimating equations. Statist Med. 2020;39(21):2779–92. 10.1002/sim.8575 32578264

[21] Elo S, Kyngäs H. The qualitative content analysis process. J Adv Nurs. 2008;62(1):107–15. doi: 10.1111/j.1365-2648.2007.04569.x 18352969

[22] SNAKS-Swedisch Network for National Clinical Studies in Obstetrics and Gynecology; 2022. Available from: https://www-snaks.se/.

