# Supplementary material for: Can the use of a next generation partograph based on WHO’s latest intrapartum care recommendations improve neonatal outcomes? (PICRINO) Study protocol for a stepped-wedge cluster randomized trial
Source: PLoS One. 2025 Mar 4;20(3):e0316336. doi: 10.1371/journal.pone.0316336 (PMC11878932; doi:10.1371/journal.pone.0316336)
Supplement: S2 File — (PDF) [file pone.0316336.s002.pdf]

Can the use of a next generation partograph based on WHO's latest intrapartum care recommendations improve neonatal outcomes? A stepped-wedge cluster randomized trial (PICRINO)

Principal Investigator and Research Team:

Principal investigator:

Marie Blomberg MD, PhD[1]

Co-Investigators:

Thomas Abrahamsson, MD, PhD[2]

Sophia Brismar Wendel MD, PhD[3]

Ylva Carlsson, MD, PhD[4]

Therese Eriksson PhD[5]

Karin Källén PhD[6]

Louise Lundborg, CNM, PhD[7]

Anna Ramö Isgren MD, PhD[1]

Katarina Remaeus MD, PhD[8]

Kristin Thomas PhD[9]

Karin Westin Mrs

Anna Karin Wikström MD, PhD[10]

Contact information:

[1]Department of Obstetrics and Gynecology; Department of Biomedical and Clinical Sciences, Linköping University, Linköping, Sweden.

[2]Department of Biomedical and Clinical Sciences, Linköping University; Crown Princess Victoria Children´s Hospital, Linköping, Sweden.

[3]Department of Obstetrics and Gynecology, Danderyd Hospital, Stockholm, Sweden; Department of Clinical Sciences, Karolinska Institutet, Danderyd Hospital, Stockholm, Sweden.

[4]Department of Obstetrics and Gynecology, Sahlgrenska University Hospital, Gothenburg, Sweden; Department of Obstetrics and Gynecology, Gothenburg Institute of Clinical Sciences, University of Gothenburg, Gothenburg, Sweden.

[5]Department of Health, Medicine and Caring Sciences, Linköping University, Linköping, Sweden.
[6]Center for Reproductive Epidemiology, Tornblad Institute, Lund University, Lund, Sweden.
[7]Clinical Epidemiology Division, Department of Medicine, Solna, Karolinska Institutet, Stockholm, Sweden.
[8]Karolinska Institutet, Stockholm, Sweden.
[9]Department of Health, Medicine and Caring, Medical Faculty, Linköping University, Linköping, Sweden.
[10]Department of Women's and Children's Health; Uppsala University, Uppsala, Sweden.

Table of Contents

Principal Investigator and Research Team: 1
Protocol summary 4
List of Abbreviations: 5
Background and Significance: 5
  The WHO Labour Care Guide 6
  Development of the Swedish Labour Care Guide (LCG-SE) 7
  Evaluation of the implementation process 8
Overall aim of the research project: 9
  Primary Objectives 9
  Secondary Objectives 9
Study design/methodology: 11
  Implementation package and support 12
  Substudy I, childbirth experience 13
  Substudy II, provider experience of LCG 15
  Substudy III, economic considerations 16
Study Population: 17
  Substudy I, childbirth experience, questionnaire and interviews 17
  Substudy II, provider experience of LCG 17
  Substudy III, economic considerations 17
  Inclusion /Exclusion Criteria 17
    Substudy I, childbirth experience, questionnaire, and interviews 18
    Substudy II, provider experience of LCG 18
Safety analysis: 18

Power analysis: 18
Ethical approval: 21
Study Timeline: 21
Conflict of Interest: 22
References: 22

Protocol summary

Study Title

Can the use of a next generation partograph based on WHO's latest intrapartum care recommendations improve neonatal outcomes? A stepped-wedge cluster randomized trial (PICRINO).

Primary Objectives

To compare two different guidelines for monitoring labor progress, the World Health Organization (WHO)'s Labour Care Guide (LCG) with standard care, and evaluate:

1) Adverse neonatal outcome, a composite outcome of perinatal and neonatal mortality and neonatal morbidity. Neonatal morbidity will include five-minute Apgar score <7, hypoxic ischemic encephalopathy II-III, and admission to a neonatal unit.
2) The rate of intrapartum cesarean section.

Secondary Objectives

Secondary outcomes are several relevant neonatal and obstetric variables, women´s and partner´s experiences of childbirth, healthcare providers experiences to LCG and economic considerations.

Study Design

A multicenter, stepped-wedge cluster randomized trial.

Study Population

All women in active labor at participating delivery units in Sweden.

Power analysis

With significance level 0.05, the power to detect the anticipated risk reduction (20%) would be >0.999.

Study Duration

2023-2025

Trial registration

The trial has been registered at www.clinicaltrials.gov: NCT05560802

Ethical approval

The Regional Ethical Review Board in Sweden approved this study on November 9th, 2022 (Dnr 2022-04868-01).

List of Abbreviations:

WHO World Health Organization

LCG Labour Care Guide

LCG-SE Swedish version of LCG

CEQ Childbirth Experience Questionnaire

CS cesarean section

SPR Swedish Pregnancy Register

SNQ Swedish Neonatal quality register

NU neonatal unit

VAS visual analog scale

Background and Significance:

Worldwide, there are current discussions regarding how surveillance and management of labor are best performed in order to optimize outcome for both the mother and the infant. New data on normal labor challenge the current definition and guidelines for labor progress in Sweden (1-3). In 2018, the World Health Organization (WHO) changed their recommendations for the definition of onset and progress of labor, (4) and in 2020 they published a new guideline for labor care, the LCG (5). The LCG includes seven sections to guide monitoring and documentation in labor. Compared with standard care guidelines used in Sweden today, the woman reaches active phase of labor at a later cervical opening, and a slower labor progress is accepted. This challenges many aspects of obstetric care since certain time limits, until now, have been a backbone in the surveillance of labor until delivery. The LCG has been developed in low- and middle-income settings, mainly for women with low-risk pregnancies, but WHO recommends use of the LCG for all pregnancies (regardless of risk) and in all formal healthcare settings. The usability and feasibility of the LCG have been tested in health facilities in South America, Asia and Africa (6). No study has however compared LCG with standard care in regard to adverse neonatal outcomes. The most severe neonatal outcomes comprising neonatal deaths and severe infant brain damage are often related to obstetric catastrophes such as uterine rupture, shoulder dystocia and abruptio placentae. Implementing LCG could affect the rate of these rare obstetric events and thereby the number of infants born with severe outcomes. On the other hand, admission to neonatal unit (NU) and neonatal resuscitation outside the delivery room could also be a traumatic event for parents. It is likely that LCG would decrease these outcomes compared with standard care.

Hence, there is a need to evaluate the impact of LCG on neonatal outcomes from both these perspectives.

More than half of all birthing women in Sweden receive oxytocin augmentation due to slow labor progress based on the current guidelines. Nevertheless, oxytocin use in labor is associated with uterine hyperstimulation with increased risk of abnormal fetal heart rate and neonatal acidemia at birth (7, 8). The rate of term intrapartum cesarean section (CS) is relatively low in Sweden (10% in 2022, all parities, induced and spontaneous labor). Although, there are differences between the delivery units (6.1-13.5%), despite all being governmentally financed. Recently, a randomized control trial from India showed a significant reduction in primary CS in the LCG group (9). No studies have evaluated the impact of LCG on intrapartum CS in a low CS setting.

The LCG entails increased individualized care, promotes women centered care, emphasizes monitoring of supportive care, and optimizes the use of interventions. Therefore, LCG might improve neonatal and maternal outcomes.

The WHO Labour Care Guide

In 2018, WHO published new recommendations on intrapartum care for a positive childbirth experience, including recommendations for care of women during labor and birth (4). The recommendations were based on evidence from systematic reviews and included updated definitions and durations for first and second stage of labor (10, 11). The duration of the first and second stage of labor is highly variable between women, and a cervical dilation rate of 1cm per hour in the first stage is unrealistically fast for some women. A cervical dilatation rate slower than 1cm per hour was by itself a poor predictor of adverse maternal and neonatal outcomes and should not be the sole indication for obstetric intervention. They concluded that older partographs (particularly those with 'alert' and 'action' lines) were no longer scientifically valid. Furthermore, many partographs in current use do not monitor supportive care interventions such as companionship, women's mobility, birth position or use of pain relief.

To facilitate implementation of these recommendations, WHO developed a "next-generation" partograph known as the WHO LCG, as well as a LCG user's manual (5). The LCG is a labor tool that supports providers in effectively monitoring maternal and fetal status, and progress of labor and offers timely reminders on appropriate clinical and supportive care. The LCG aims to promote women centered care, stimulate providers to think critically around labor

decision-making, and individualize labor monitoring. The LCG was developed through several expert consultations, iterative prototype development and testing, an international survey of maternity care providers, and qualitative research with midwives from six African countries. Following its publication in December 2020, a six-country evaluation project explored the LCG's usability, feasibility, and acceptability in different settings, as well as barriers and facilitators to its use (6, 12). Using the LCG for managing labor and birth is now the WHO recommended standard for providing intrapartum care internationally.

The LCG includes assessments and observations that are essential for the care of all pregnant women, regardless of their risk status or where they give birth (i.e., high-resourced, or limited-resourced facility). Embedded in the LCG are parameters and alerts to facilitate the use of interventions known to reduce the use of unnecessary augmentation, minimize the need for cesarean section (CS), and improve birth experiences. While the LCG was primarily designed to be used for the care of healthy pregnant women (i.e., women with low-risk pregnancies), women classified as risk when attending the labor ward can still benefit from the LCG as a monitoring tool (13).

Development of the Swedish Labour Care Guide (LCG-SE)

With support from the Swedish Research Council (grant nr 2021-06573) the Swedish Labour Care Guide (LCG-SE) has been systematically developed by health care professionals in maternity care in Linköping, Sweden. The aim was to systematically modify and adapt a version of the WHO´s labour Care Guide (LCG) to a Swedish setting. The developed protocol was intended to be used in the national trial (PICRINO). In early 2022 a local steering group was established to develop a Swedish version of the WHO´s LCG. The group consisted of clinicians working in the labor ward at the University Hospital in Linköping including obstetricians, midwifes (at different levels of experience) and assistant nurses. They were all educated in the WHO´s LCG and the LCG manual was translated into Swedish. Four structured workshops with the health care professionals working in the labor ward in Linköping were conducted. Each workshop lasting three hours and was led by two to three members of the steering committee. Participation was voluntary. After completion, the members of the steering group reviewed the protocol, based on input from the workshops, with the aim to identify elements to adapt. The blueprint of a modified protocol was then tested by case discussions by both local and national clinicians, resulting in some minor further adjustments. Overall, the LCG-SE has undergone moderate adaptations compared with

the WHO´s LCG. No changes have been made of the clinical recommendations embedded within LCG (including durations of labor or triggers for intervention).

Conditions for implementing LCG-SE in delivery care in terms of perceived acceptability, feasibility, appropriateness, and usability among health care professionals will be explored in October 2022. We estimate that about five focus groups with 4-6 informants per group will be sufficient to address the study aim. This estimation is based on recruitment strategy and data collection methods. Approximately 25 midwives and doctors working in Linköping (University hospital) and Norrköping (secondary level hospital) have signed up to test the LCG-SE in daily clinical practice for three weeks, although without changing any clinical guidelines. Explorative qualitative focus group interviews with key stakeholders of the implementation will be held after these three weeks to capture how informants discuss and reason around the use of LCG-SE in practice. The groups will consist of a mixture of professions to promote multi-professional discussions and exchange. A semi-structured interview guide will be used during interviews and include open questions on the perceived acceptability, feasibility, appropriateness, and usability of LCG-SE in practice as well as how the new way of working could influence work climate. An illustration of the LCG-SE will be used in interviews. All interviews will be audio recorded and transcribed verbatim. An explorative approach will be used employing inductive content analysis according to Elo and Kyngäs (14). The Consolidated criteria for reporting qualitative research will be used during data collection and analysis as well as reporting of results.

Evaluation of the implementation process

An implementation process is complex and depends on numerous of factors at different levels within the system. Failure in adherence to interventions is a broad problem and a successful implementation process is affected by the organization's readiness. The concept organizational readiness for change (ORC) is two-dimensional referring to member's motivation/willingness and capability to implement a change in practice. To measure and consider ORC during an implementation process may facilitate the process and can be used to explain outcomes in fidelity. Further, to reduce the risk of evaluating an intervention that has not been adequately implemented, it is also crucial to evaluate the implementation fidelity in participating sites in implementation studies (15). Therefore, these factors are important to consider in the evaluation of the implementation of LCG in the PICRINO trial.

Overall aim of the research project:

The overall aim is to evaluate the impact of the use of two different guidelines for monitoring labor progress, the WHO´s LCG versus standard care, on neonatal and maternal outcomes. The hypothesis is that the use of LCG will reduce adverse neonatal outcomes and decrease the number of intrapartum CS compared with standard care.
Secondly, other perinatal interventions and complications will be compared between the LCG and standard care groups, as well as economic considerations. This will be investigated using a multicenter, stepped-wedge cluster randomized trial design.
In addition, the project will explore a series of quantitative and qualitative research questions to gain in-depth knowledge about experiences and perceptions about childbirth and the use of LCG. These research questions will be investigated using questionnaires, focus group and individual interviews with providers, partners and women that have gone through childbirth.

Primary Objectives

To compare two different guidelines for monitoring labor progress, the WHO´s LCG with standard care and evaluate:

1) Adverse neonatal outcome, a composite outcome of perinatal and neonatal mortality and neonatal morbidity. Neonatal morbidity will include five-minute Apgar score <7, hypoxic ischemic encephalopathy II-III and admission to a NU.
2) The rate of intrapartum cesarean section.

Secondary Objectives

Table 1 lists the secondary outcomes and topics that will be investigated within the trial.

**Secondary outcomes/topics**	**Definitions/data sources**
Neonatal outcomes	
Five-minute Apgar score <7	
Admission to neonatal unit	
Hypoxic ischemic encephalopathy II-III	ICD-10 code
Intracranial hemorrhage	ICD-10 code
Neonatal seizures	ICD-10 code
Meconium aspiration syndrome	ICD-10 code
Five-minute Apgar score <4	
Perinatal (number of stillbirths after 22 weeks gestation and deaths in the first week	

of life) and neonatal mortality (during the first 28 days of life)	
Respiratory disorders	ICD-10 code
Neonatal infection	ICD-10 code
Neonatal hypoglycemia	ICD-10 code
Neonatal jaundice	ICD-10 code
Shoulder dystocia	ICD-10 code
Obstetric brachial plexus injury	ICD-10 code
Obstetric outcomes	
Rates of spontaneous vaginal delivery	
Rates of instrumental delivery	
Indications for intrapartum cesarean section	
Rate of artificial rupture of membranes	
Rates of oxytocin use for augmentation of labor	
Cervical dilation at onset of augmentation	LCG
Adherence to oxytocin use recommendations (fidelity)	LCG
Rates of epidural use	
Rates of postpartum hemorrhage >1000ml	
Amount of postpartum bleeding, ml	
Rates of perineal laceration (grade II-IV)	ICD-10 code
Duration of labor	Time estimates of active phase of labor
Duration of labor	Time estimates of second stage of labor
Factors potentially related to changes in obstetric care during the study period	
Rates of elective cesarean section	
Rates of induction of labor	
**Substudy 1 - Childbirth experience (women and partners)**	
Women´s experiences of childbirth	Assessment of childbirth experience by a visual analog scale (VAS)

Women´s experiences of childbirth	Questionnaires distributed by the SPR
Women´s experiences of childbirth	Questionnaire, CEQ2
Women´s experiences of childbirth	Individual interviews
Partner´s experiences of childbirth	Individual interviews
**Substudy II – Experience of LCG (providers)**	
Perceptions of organizational readiness for implementing LCG	Questionnaire, E-Ready
Experiences and perceptions about using LCG in practice	Questionnaire, focus group interviews
**Substudy III - Economic evaluation (PICRINO)**	
What is the cost of LCG compared to standard care regarding healthcare utilization for women and children?	Regional patient register
How is the need for providers and premises affected by the introduction of LCG compared to standard care?	Regional patient register

Outcome variables will be extracted from two Swedish Quality Registers. The Swedish Pregnancy Register (SPR) is a rank 1 certified national quality register containing prospectively collected data from early pregnancy to a few months after birth and the Swedish Neonatal Quality Register (SNQ) which includes data on neonatal outcomes for all infants admitted to neonatal care. Data on healthcare utilization, DRG and cost per patient will be obtained from each region's patient register. A linkage between the registers will be done by the National Board of Health and Welfare and a dataset without personal identification possibilities will be distributed to the research team.

Study design/methodology:

This is a multicenter, stepped-wedge cluster randomized trial (16). The intervention LCG will be introduced over a 24-month period 2023-2024. Data will be collected over a 24-month period. Data in the 'active phase' after introduction of the intervention LCG (dark blue, figure1) will be compared with data in the 'control phase'—the period during which standard care is

followed, from trial start to the time of introduction of the intervention (light blue, figure 1). A transition period (white, figure 1) of two month between introduction of the intervention and data outcome collection will be used.

Figure 1.

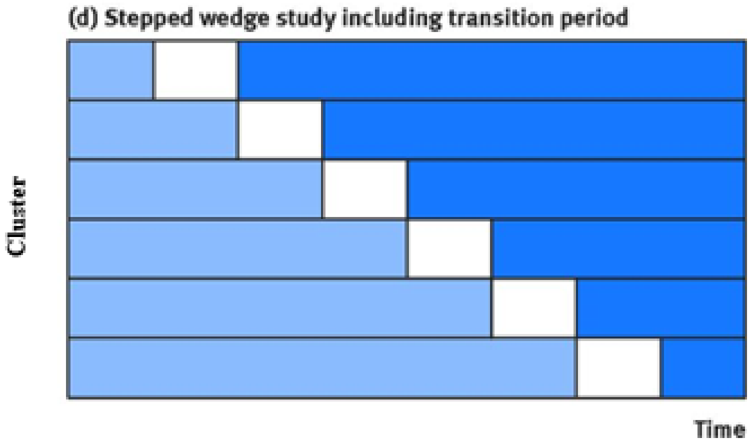

Implementation package and support

All included sites will undergo a structured implementation package with the LCG. The implementation will start at each site at the beginning of the wash-out period according to the time-schedule for the trial. The education in LCG will be provided by a specially trained group of midwives and obstetricians (the LCG implementation group).

Key questions at the digital recruitment meetings held with all interested study sites were to assure that the management team was positive and supportive and that the staff had expressed curiosity of participating in PICRINO. Both members from the LCG implementation group and researchers participated in those meetings to cover all putative questions raised.

Furthermore, the LCG implementation group has identified that supportive supervision from senior staff is crucial. Each site will have one or more local appointed providers (midwife and/or obstetrician) that will have regular contact (phonecalls, e-mails, online meetings) with the LCG implementation group. Each site will also have a local appointed administrative staff to facilitate the administrative work that the implementation entails. About 3-4 months prior to the implementation at each site the appointed providers and administrative staff will be offered a one-day training about LCG at site in Linköping.

Prior to the introduction, members of the LCG implementation group will participate in at least one staff meeting at each site to give a short introduction to LCG and to answer

questions about LCG. At the start day of the transition period, all providers (assistant nurses, midwifes and physicians) will receive an introduction package including power points, recorded information materials and written information about LCG, background of the evidence and instructions on documentation in the document LCG. About two weeks later, all providers will undergo a four hours training including a two-hour lecture about LCG followed by workshops with pre-designed case descriptions where providers will practice the LCG guidelines and learn how to document in the LCG protocol. This training will be held at the local sites. The providers will have a detailed user´s manual to their help during the learning phase and a quick user´s manual as a support along the trial. After the four hours training shorter sessions will be held, 1-2 times/week the following four to six weeks, where providers will have the opportunity to ask questions and practice the LCG protocol by case-based learning with real world cases. The sessions will be held both at the local sites and as online meetings. The new routine with LCG will also be communicated to the staff orally at regular daily meetings, with posters and e-mails. After four to six weeks, the LCG implementation group will ensure that all staff members have received adequate training and that no old partographs are used. Further, regularly follow ups during the trial period will be held to refresh the information and educate new staff.

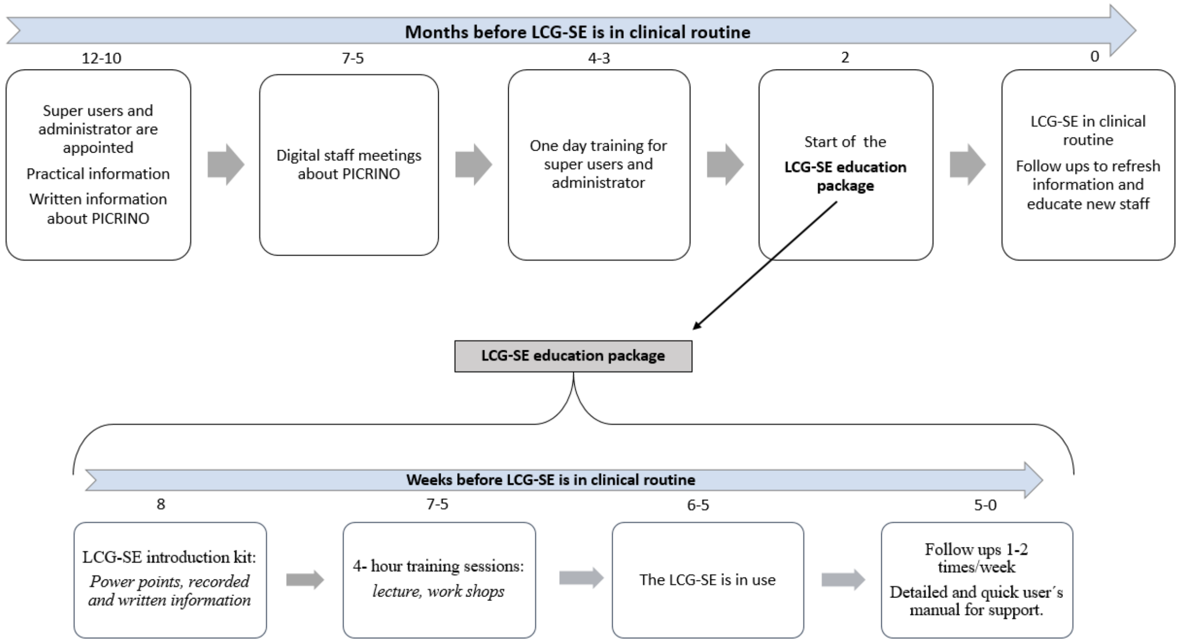

Figure 2. The proposed implementation process for LCG-SE.

Substudy I, childbirth experience

Childbirth experience will be investigated using standard clinical assessments of childbirth experience, validated questionnaires, and individual interviews.

a) After delivery, women in Sweden are asked by the midwife at the postnatal ward to assess their overall experience of childbirth by using a visual analog scale (VAS). This assessment of satisfaction of childbirth by VAS is a well-established routine in the postnatal care. The VAS scoring is usually completed within 2 days after delivery, before discharge from the postnatal ward and is documented in the women's electronic medical record. Categorized VAS scores will be compared between women giving birth during standard care versus LCG-SE.

b) Questionnaires administered by the SPR are distributed to women eight weeks and one year after childbirth through an account at the Swedish 1177.se. These questionnaires are extensive and cover all parts of the pregnancy, delivery and the postpartum period. For the purpose of the present study selected questions concerning childbirth experience will be used. These questionnaires are available in English, Arabic, Farsi, Finnish, French, Spanish and Somali. Comparison between women giving birth during standard care versus LCG will be performed.

c) Four weeks postpartum questions about birth experience will be randomly distributed to women at three to four participating sites with different levels of care. After electronic (using the Swedish Bank-ID) informed consent is obtained women will be linked to the validated Childbirth Experience Questionnaire (CEQ), which will be used to measure childbirth experience (17). The CEQ consists of 22 items related to childbirth experience, which are categorized into four domains: own capacity, professional support, perceived safety, and participation. The total CEQ scores, as well as scores for all four domains will be compared between women giving birth during standard care versus LCG.

d) To explore experiences of childbirth, women will also be invited to take part in individual interviews. Invitations and information about the interviews will be posted to women. A purposive sampling strategy will be used whereby we will strive to recruit a broad range of women based on e.g., age, number of children, geographic location and socioeconomic position. Women register their interest to participate by contacting the contact person for the study (as specified in the invitation and information letter). Informed consent will be made verbally prior to each interview. The procedure for informed consent will be described for participants in the participant information letter. All interviews will take place in an undisturbed setting which will be decided together with the participant. The interviews can be conducted in-person, digital meeting (audio only) or by phone depending on the preference of the

participant. An interview guide including open-ended questions to explore perceptions and experiences of childbirth will be used. Follow-up questions and probes will also be used to clarify and deepen the understanding of the participants' responses. The participants will be able to, at any time and without having to give a reason, withdraw from the interview and the participation in the study. All interviews will be audio-recorded and expected to be about one hour in duration, field notes will be written by the interviewer after each interview. The audio-recordings will be transcribed verbatim by a professional transcribing service and each transcript will be assigned an anonymous code to safeguard the confidentiality of the respondent. Inductive content analysis according to Elo & Kyngäs (14) will be used in data analysis. We estimate that approximately 20-25 interviews will be adequate to capture the study aim based on the research question and analysis method.

e) To further explore childbirth experiences, interviews with partners will also be carried out. Recruitment strategy, eligible criteria, data collection and analysis methods will follow the same procedures as interviews with women described above. To clarify, however, partners will also be invited to take part in interviews through a postal letter which will also include information about the study. Verbal informed consent will be made prior to each interview and about 20-25 interviews are estimated to be adequate to capture study aims.

Substudy II, provider experience of LCG

Provider experience and perceptions about using LCG will be investigated using focus group interviews and validated questionnaires.

a) Provider perception of organizational readiness for implementing LCG will be measured during the transition period. After the introduction, where all staff have received training about LCG, the validated tool E-Ready (18), will be e-mailed by a web-link to nurse assistants, midwives and physicians at selected study sites. E-Ready consists of 6 sections with sub items investigating perceived conditions for change at workplace, perceived individual conditions for change, perceived support and engagement among management, perceived readiness among colleagues, perceived consequences on status quo and perceived workplace attitudes. In addition, 7 single items investigating compatibility with current work routines, commitment to change and some background questions on respondents are included. E-Ready takes approximately 10 minutes to complete. Minor modifications will be made in the

original tool to fit the purpose of this study. Informed consent to distribute questionnaires will be obtained from each hospital administrator and written participant information will be sent along with the questionnaire to the providers.

b) Provider experience of working with LCG will be investigated using focus group interviews. Providers will be invited to take part in focus group interviews via e-mail which also include information about the study. Focus group interviews are deemed appropriate since the implementation of LCG is team based and this method would enable inter-professional interaction and discussion about implementation experiences. We estimate that 4-6 focus groups with about six informants per group will be sufficient to capture rich data on implementation. Similar to earlier phases of the project, focus groups will consist of a mixture of professions to promote multi-professional discussions and exchange. Written informed consent will be made at the focus group, prior discussion and recording starts. Participants will give their consent by signing form attached to the participant information letter. A semi-structured interview guide will be employed and aim to capture experiences, perceptions and expectations of LCG as well as hinders and facilitators for implementation. All interviews will be audio recorded and transcribed verbatim. An explorative approach will be used employing inductive content analysis according to Elo and Kyngäs (14).

c) A questionnaire evaluating usability and satisfaction among health care providers will be used. The questionnaire will be sent to enrolled clinics and their health care personnel working at the labour ward about three months after the clinic has started using LCG-SE.

Substudy III, economic considerations

Data on health care utilization for both women and children, during labor but also during the following year will be collected. Data on healthcare consumption is collected regardless of level of care and therefore includes primary, outpatient and inpatient care. Data on healthcare utilization, DRG and cost per patient are obtained from each region's patient register and will be interlinked with data from the SPR and SNQ.

The introduction of LCG may affect the time in labor and thus the logistics of how providers and rooms need to be scheduled. In order to analyze the effects from a logistic perspective, the occupancy rate and provider structure at each unit will be collected.

Study Population:

All 46 different delivery units in Sweden has received an invitation to participate in the trial (115000 deliveries annually). The number of childbirths at each unit ranges from 500 to 12000 (which will be considered in the stepped-wedge inclusion process). Each center will contribute with both cases and controls according to the proposed study design. Primary outcome will be determined among women with term singleton pregnancies and cephalic presentation.

Substudy I, childbirth experience, questionnaire and interviews

Childbirth experience will be evaluated in the total study population by clinically determined VAS estimates and by questionnaires administered by the SPR. Further, randomly selected women at three to four hospitals with different levels of care, approximately 1000 questionnaires in total. With an expected response rate of 50%, 2000 questionnaires will be distributed. For individual interviews, we estimate that approximately 20-25 interviews will be adequate to capture the study aim. This is based on the nature of the research question, interview guide, estimated duration of the interviews and analysis methods.

Substudy II, provider experience of LCG

To gain a broad understanding of the perceptions of organizational readiness for implementing LCG, study-sites will be selected based on variation in organization, geographical spread and teaching status. About 10 study sites with an estimation of 1500 employees will be invited. We appraise that about 4-6 focus groups interviews (with approximately six individuals in each group) is adequate to capture the study aim. This is based on the nature of the research questions, interview guide, estimated duration of the interviews and analysis methods. The questionnaire concerning provider experience of LCG will be sent to all health care workers at the enrolled clinics. The preliminary number of questionnaires will be about 3400.

Substudy III, economic considerations

All included study sites

Inclusion /Exclusion Criteria

Inclusion criteria is all women in active labor at participating sites. Guidelines and local recommendations concerning complicated pregnancies and deliveries will not be changed and should be followed accordingly during the study period. No exclusion criteria.

Substudy I, childbirth experience, questionnaire, and interviews

Inclusion criteria; women who have been in active labor. Exclusion criteria for both questionnaire and interview data will be twin pregnancy, preterm delivery, breech delivery, not able to speak and read Swedish and younger than 18 years.

Substudy II, provider experience of LCG

Eligible criteria will be experience of using LCG in clinical practice. Exclusion criteria for questionnaire will be providers employed for less than three months at the labor ward.

Safety analysis:

Two interim analyses will be performed one-third and two-thirds of the way through the trial. The interim analyses are focused on safety and will be performed by comparing the primary core outcome set (perinatal and neonatal mortality, 5-minute Apgar score <7, occurrence of hypoxic ischemic encephalopathy grades II–III, and admission to a neonatal unit) in the control group (standard care) and the intervention group (LCG) by a statistician who is not involved in the PICRINO trial. Neonatal outcomes will be extracted from the SPR and SNQ. A Security Board that includes the external statistician and an experienced obstetrician, as well as a neonatologist will interpret the data. These individuals will all be independent of the PICRINO Steering Committee. Furthermore, the PICRINO Steering Committee will be kept blinded to the results unless a 'stop' recommendation is made by this independent Security Board based on a statistically significant clinically worse outcome ($p<0.05$) in the intervention group. The study will not be interrupted due to a significantly better clinical outcome in the intervention group compared with the control group since the new guidelines could not be implemented faster than via the PICRINO trial protocol.

Power analysis:

Power analyses were performed considering the number of time periods, number of clusters, observations per time period, the before trial incidence of outcome, the between-units-variation coefficient, and anticipated risk reduction.

The incident of any adverse neonatal outcome (defined as perinatal or neonatal death, Apgar score <7 at five minutes, occurrence of hypoxic ischemic encephalopathy grade II-III, or admission to a NU) was estimated using data from the SPR and the SNQ (Figure 3)

Number of time periods: All deliveries during a period of two years divided into 7 time periods.

Number of clusters: Cautiously, it is assumed that half of the 44 Swedish delivery units will participate in the trial (thus, 22 units).

Number of observations per time period: The total number of births that met the inclusion criteria during the five observed years shown in Figure 3 was N= 466 615, giving a yearly estimate of N=93 300 births meeting the inclusion criteria, consistent with approximately 15 000 observations (2*93 300/12) per time unit. With the cautious estimation that half of the delivery units will participate, we will reach approximately 7500 observations per time unit.

Pre-trial mean incidence of composite outcome: 6.73% (Figure 3).

Between unit coefficient of variation: 0.46 (Figure 3, variance between units / overall mean)

Anticipated risk reduction: 20% Risk reduction (corresponding to a Risk Ratio of 0.8).

Power: With significance level 0.05, the power to detect the anticipated risk reduction would be >0.999 given the settings listed above.

Figure 3. Incidence of adverse neonatal outcome[A] by delivery unit (with 95% confidence intervals) including term singletons in cephalic presentation. Elective cesarean sections were excluded. Sources: The Swedish Pregnancy Register and the Swedish Neonatal Quality Register, 2017-2021.

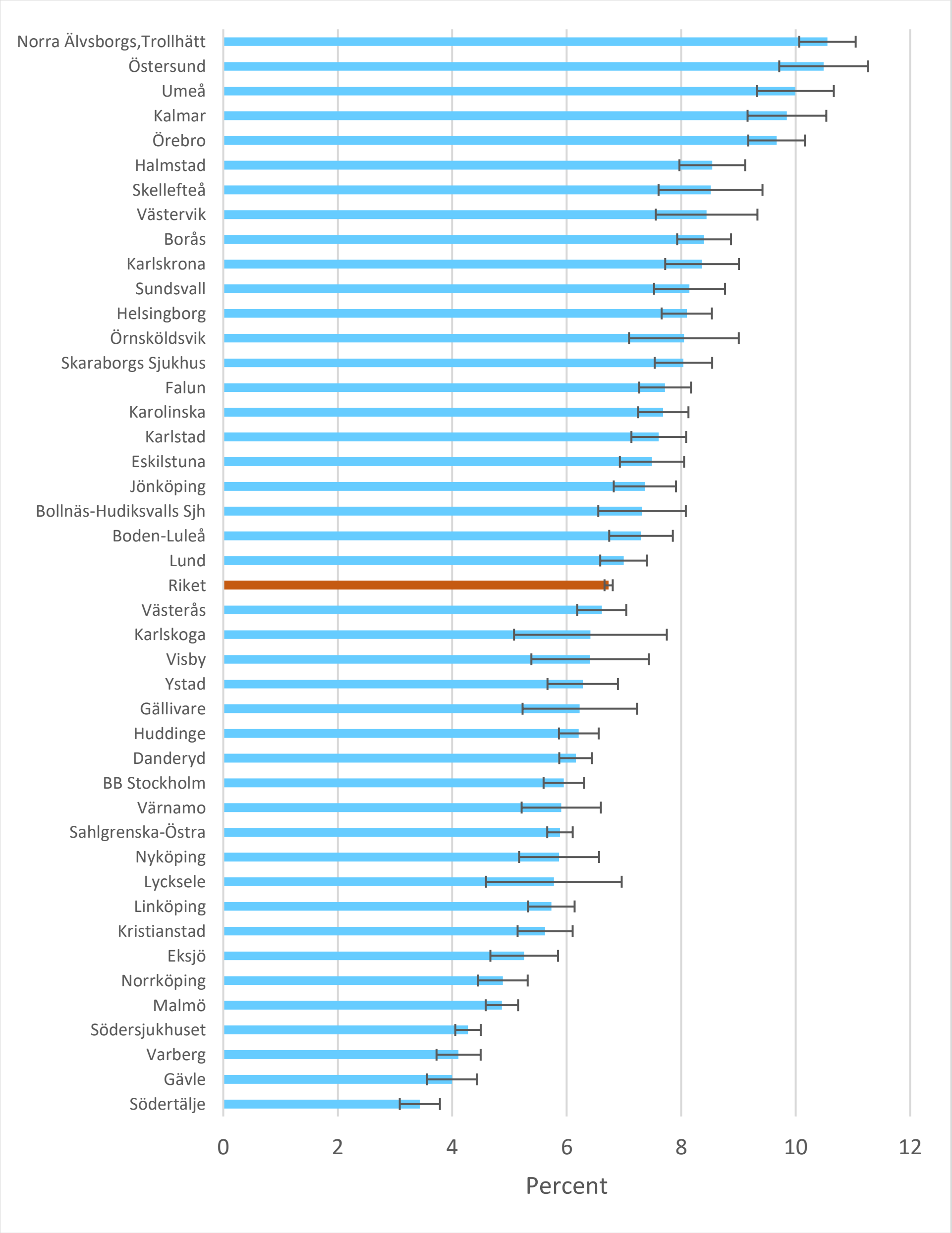

[A]Perinatal or neonatal death, Apgar score <7 at five minutes, occurrence of hypoxic ischemic encephalopathy grade II-III, or admission to neonatal unit

The rate of intrapartum CS among term pregnancies in Sweden between 2017 and 2021 was 9.1%, ranging from 5.6% to 11.8% (Figure 4). No power analysis was performed explicit for intrapartum CS (due to the higher occurrence of intrapartum CS than the incidence of the composite adverse neonatal outcome).

Figure 4 Prevalence of intrapartum cesarean sections in term pregnancies by delivery unit.

Source: The Swedish Pregnancy Register, 2017-2021.

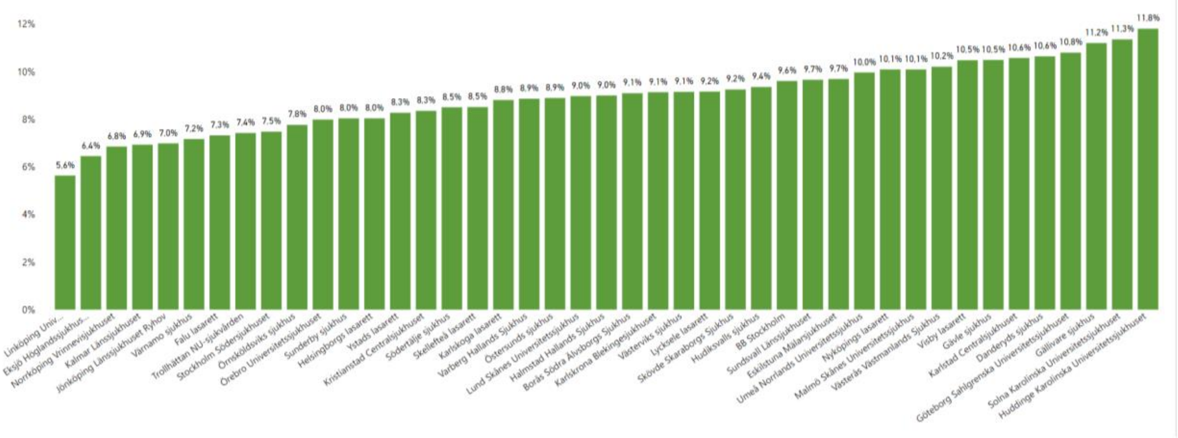

Ethical approval:

The regional Ethical Review Board in Sweden approved this study on November 9th, 2022 (Dnr 2022-04868-01).

Study Timeline:

**Activity**	**2022**		**2023**		**2024**	**2025**	**2026**
	**Q3**	**Q4**	**Q1-Q2**	**Q3-Q4**	**Q1-Q4**	**Q1-Q4**	**Q1-Q4**
Finalize research plan and trial protocol	X						
Application to the Ethical Review Board	X						
Validation of the Swedish version of the LCG	X	X					
Establish contact with lead obstetricians and midwives at all delivery units in Sweden	X						
Identify local key persons and obtain site participation	X	X	X				
Register the Trial registration at www.clinicaltrials.gov		X					
Disseminate information and study protocol to participating sites				X	X	X	
Enrolment of study participants				X	X	X	
Questionnaire concerning organizational readiness for implementing LCG					X		
Questionnaire concerning childbirth experience					X		
Qualitative study concerning childbirth experience					X		
Extraction and analysis of data, main study							X
Extraction and analysis of data, economical evaluation							X

Prepare and publish first results in the form of original articles in peer-reviewed scientific journals							X

Detailed timeline for the project starting 2024

**Activity** (The main trial in **bold letters**)	**2024**				**2025**			**2026**		**2027**	
	**Q1**	**Q2**	**Q3**	**Q4**	**Q1**	**Q2**	**Q3-Q4**	**Q1-Q2**	**Q3-Q4**	**Q1-Q2**	**Q3-Q4**
Evaluation of organizational readiness for implementing LCG-SE	x	x									
**Implementation of the LCG-SE, Cluster number**	**1**	**2**	**3**	**4**	**5**	**6**					
**Safety analysis (interim analysis)**			**x**		**x**						
Childbirth experience, questionnaire			x				x				
Womens' and partners´ experiences of childbirth, qualitative interviews, and analysis of data				x	x						
Perceived acceptability, feasibility, and appropriateness of the LCG-SE and its implementation process		x	x	x							
Satisfaction and perceived usability of the LCG-SE		x	x	x	x	x					
**Extraction of data for primary and secondary outcomes**								**x**	**x**		
**Analysis of data for primary and secondary outcomes**									**x**	**x**	**x**
Degree of fidelity to the LCG-SE									x	x	
Health economics evaluation									x	x	x
Presentation of results at national and international conferences										x	x
Preparation and publishing of the results in peer-reviewed scientific journals										x	x
Dissemination of results to expectant mothers, maternity wards, and policymakers										x	x

Conflict of Interest:

No authors report any conflict of interest.

References:

1. Zhang J, Landy HJ, Branch DW, Burkman R, Haberman S, Gregory KD, et al. Contemporary patterns of spontaneous labor with normal neonatal outcomes. Obstet Gynecol. 2010;116(6):1281-7.

2. Oladapo OT, Souza JP, Fawole B, Mugerwa K, Perdona G, Alves D, et al. Progression of the first stage of spontaneous labour: A prospective cohort study in two sub-Saharan African countries. PLoS Med. 2018;15(1):e1002492.

3. Lundborg L, Aberg K, Sandstrom A, Discacciati A, Tilden EL, Stephansson O, et al. First stage progression in women with spontaneous onset of labor: A large population-based cohort study. PloS one. 2020;15(9):e0239724.

4. WHO. WHO recommendations. Intrapartum care for a positive childbirth experience. WHO guidelines approved by the guidelines review committee. https://www.who.int/reproductivehealth/publications/intrapartum-care-guidelines/en/2018

5. WHO. WHO labour care guide user´s manual Geneva: World Health Organization; 2020. Report No.: Licence: CC BY-NC-SA 3.0 IGO.

6. Vogel JP, Comrie-Thomson L, Pingray V, Gadama L, Galadanci H, Goudar S, et al. Usability, acceptability, and feasibility of the World Health Organization Labour Care Guide: A mixed-methods, multicountry evaluation. Birth. 2021;48(1):66-75.

7. Burguet A, Rousseau A. Oxytocin administration during spontaneous labor: Guidelines for clinical practice. Chapter 6: Fetal, neonatal and pediatric risks and adverse effects of using oxytocin augmentation during spontaneous labor. J Gynecol Obstet Hum Reprod. 2017;46(6):523-30.
8. Mussi S, Incerti M, Plevani C, Ghidini A, Pezzullo JC, Locatelli A. Effect of oxytocin during labor on neonatal acidemia. J Matern Fetal Neonatal Med. 2016;29(19):3098-103.
9. Oladapo OT, Diaz V, Bonet M, Abalos E, Thwin SS, Souza H, et al. Cervical dilatation patterns of 'low-risk' women with spontaneous labour and normal perinatal outcomes: a systematic review. Bjog. 2018;125(8):944–54.
10. Pandey D, Bharti R, Dabral A, Khanam Z. Impact of WHO Labor Care Guide on reducing cesarean sections at a tertiary center: an open-label randomized controlled trial. AJOG Glob Rep. 2022;20:2(3):100075.
11. Abalos E, Oladapo OT, Chamillard M, Díaz V, Pasquale J, Bonet M, et al. Duration of spontaneous labour in 'low-risk' women with 'normal' perinatal outcomes: A systematic review. Eur J Obstet Gynecol Reprod Biol. 2018;223:123–32.
12. Hofmeyr GJ, Bernitz S, Bonet M, Bucagu M, Dao B, Downe S, et al. WHO next-generation partograph: revolutionary steps towards individualised labour care. BJOG. 2021;128(10):1658–62.
13. Brizuela V, Leslie HH, Sharma J, Langer A, Tunçalp Ö. Measuring quality of care for all women and newborns: how do we know if we are doing it right? A review of facility assessment tools. Lancet Glob Health. 2019;7(5):e624-e32.
14. Elo S, Kyngäs H.The qualitative content analysis process. J Adv Nurs. 2008;62:107-15.
15. Weiner BJ, Amick H, Lee SY. Conceptualization and measurement of organizational readiness for change: a review of the literature in health services research and other fields. Med Care Res Rev. 2008;65(4):379-436.
16. Hemming K, Haines TP, Chilton PJ, Girling AJ, Lilford RJ. The stepped wedge cluster randomised trial: rationale, design, analysis, and reporting. BMJ. 2015;350:h391.
17. Dencker A, Taft C, Bergqvist L, Lilja H, Berg M. Childbirth experience questionnaire (CEQ): development and evaluation of a multidimensional instrument. BMC Pregnancy Childbirth. 2010;10:81.
18. Dannapfel P, Thomas K, Chakhunashvili A, Melin J, Trolle Lagerros Y. A Self-help Tool to Facilitate Implementation of eHealth Initiatives in Health Care (E-Ready): Formative Evaluation. JMIR Form Res. 2022;17:6(1):e17568.
